# Cardiac Self-Efficacy Improvement in a Digital Heart Health Program: Secondary Analysis From a Feasibility and Acceptability Pilot Study

**DOI:** 10.2196/60676

**Published:** 2025-04-24

**Authors:** Kimberly G Lockwood, Priya R Kulkarni, OraLee H Branch, Sarah A Graham

**Affiliations:** 1 Clinical Research Department, Lark Health, 809 Cuesta Dr. Suite B #1033, Mountain View, CA, 94040, United States, 1 650-300-1755; 2 Digital Health Technologies Department, Roche Information Solutions, Santa Clara, CA, United States

**Keywords:** digital health, self-efficacy, behavior change, prevention, cardiovascular health, digital heart health, lifestyle, heart health, health coaching, mobile phone

## Abstract

**Background:**

Lifestyle modification programs play a critical role in preventing and managing cardiovascular disease (CVD). A key aim of many programs is improving patients’ self-efficacy. In-person lifestyle modification programs can enhance self-efficacy in managing CVD risk, also known as cardiac self-efficacy (CSE). However, such programs are typically staffing and resource intensive. Digital lifestyle modification programs may offer a scalable and accessible way to improve CSE, but this has not been shown in prior research.

**Objective:**

This study examined changes in CSE among individuals using a digital lifestyle modification program for cardiovascular health. Evaluation of improvement in CSE was a secondary goal of a feasibility and acceptability pilot study of a digital program for Heart Health.

**Methods:**

Participants were individuals with elevated risk for CVD who enrolled in a 90-day pilot study that involved mobile app–based, artificial intelligence–powered health coaching and educational lessons focused on behaviors that promote cardiovascular health. Participants completed the 9-item CSE Scale at baseline and in month 2. Changes in confidence in participants’ ability to manage their cardiovascular health were assessed.

**Results:**

The sample included 273 (n=207, 61.2% female; mean age 59.3, SD 10.1 years) participants who submitted a complete CSE Scale at baseline and in month 2. The total CSE Scale score increased by 12.9% (*P*<.001) from baseline to month 2. Additionally, there were significant increases in mean score on each of the 9 individual CSE Scale items (all *P*<.001), with the largest increases in confidence “in knowing when to call or visit the doctor for your heart disease” (17% increase; *P*<.001), “in knowing how much physical activity is good for you” (16.3% increase; *P*<.001), and “that you can get regular aerobic exercise” (19% increase; *P*<.001).

**Conclusions:**

The present analyses indicate that participants in a digital lifestyle modification program for cardiovascular health showed significant improvements in CSE within 2 months. This work adds to the growing literature examining ways to improve health-related self-efficacy and scalable access to programs for prevention and management of CVD.

## Introduction

### Background

Cardiovascular disease (CVD) is the leading cause of death and the most common chronic disease in the United States, with 126.9 million adults (nearly 50% of the population) living with some form of CVD [1]. CVD is a key public health concern, as well as an economic burden, with costs related to CVD estimated to be US $378 billion, exceeding all other chronic health conditions [1,2]. Prevention and risk reduction for patients at risk for CVD is important to reduce mortality, disability, and economic burden [3]. According to the American Heart Association, engaging in a healthy lifestyle over the course of one’s lifespan is the most important factor in CVD prevention [4]. As maintaining a healthy lifestyle is difficult for many adults [5], 1 method of CVD prevention and management is participation in behavioral interventions focused on healthy lifestyle modifications [4]. Position statements from the US Preventive Services Task Force state that lifestyle modification programs that emphasize a healthy diet and physical activity have a wide variety of cardiovascular health benefits and CVD risk reduction among individuals with and without a diagnosis of CVD [6-8]. To enable individuals to make lasting behavior changes to improve their cardiovascular health, a key aim of many lifestyle modification programs is improving patients’ self-efficacy to manage their health [7,9,10].

### Importance of Self-Management in CVD

Self-efficacy, or confidence in one’s ability to execute and control certain behaviors, is crucial for patient empowerment and self-management of cardiovascular health [11]. There are several evaluation tools for measuring self-efficacy in the context of disease self-management. For instance, general scales for managing chronic disease include the Chronic Disease Self-Efficacy Scale [12] and the Self-Efficacy for Managing Chronic Disease 6-Item Scale [13]. The Cardiac Self-Efficacy (CSE) Scale is a measure of self-efficacy specifically for self-management of CVD [14]. The CSE Scale is a self-report survey that evaluates individuals’ beliefs in their ability to manage their cardiovascular health and the challenges that come with heart disease [14,15]. These self-efficacy evaluation tools provide insight into how confident an individual feels about carrying out behaviors that are beneficial to disease prevention and self-management.

### Self-Efficacy in CVD Prevention and Management

Self-efficacy is a key target of lifestyle modification programs because it predicts a range of healthy behaviors and outcomes among individuals with CVD. For instance, higher self-efficacy is associated with greater engagement in self-care behaviors [16,17], such as higher levels of physical activity [18], healthier dietary habits [19], and better medication adherence [20]. Among cardiac patients, higher self-efficacy is also associated with better attendance in cardiac rehabilitation programs [21]. Meta-analytic results also indicate that stronger self-efficacy among those with CVD is linked with a range of metrics of health-related quality of life, such as physical functioning and mobility, emotional functioning, mental health, and social functioning [22].

A review of the literature indicated that in-person lifestyle modification programs for CVD have been successful in improving health-related self-efficacy [10]. One program involved 6 weeks of in-person, 2.5-hour educational sessions led by trained peer leaders who received 4 days of training; results of this study showed significant improvements in self-efficacy related to managing their health [23]. Another program that included 8 hour-long, individual sessions between each patient and a health professional found significant increases in self-efficacy related to self-care after 12 weeks [24].

There is also evidence that in-person lifestyle modification programs can improve self-efficacy specific to managing cardiovascular health, as measured by the CSE Scale. Recent studies show that intensive, in-person lifestyle modification programs for CVD led by nurses and health professionals can improve CSE scores over time [25,26], which have been shown to lead to downstream improvement of health behaviors and outcomes over time [14,27,28].

The evidence reviewed here shows that, although in-person programs have succeeded in improving self-efficacy, these intensive programs typically involve a high degree of human contact and staffing resources; this makes them difficult to implement or impossible to scale up to larger populations. Moreover, in-person programs can present access challenges to patients, such as having to travel long distances to attend [21]. As such, scalable and accessible lifestyle modification programs to improve CSE are needed.

### Digital Health and CSE

Recent years have seen an increase in the availability of digital lifestyle modification programs for CVD prevention and management [29-31]. These digital health solutions have become increasingly available and can provide many benefits over in-person programs, such as increased accessibility, on-demand support, availability outside of typical working hours, lower cost, and scalability to larger populations [32]. These benefits have the potential to increase participation in lifestyle modifications for CVD [30]. Moreover, digital health programs for CVD prevention and management can significantly improve patient behaviors and show promise in delivering care that is accessible, cost-effective, and patient-focused [33].

Despite these benefits, many outcomes including CSE, remain unexplored in digital lifestyle modification programs and more evidence is required [31]. There is some evidence that digital interventions can increase general self-efficacy [34,35], suggesting that it may be possible for digital CVD programs to improve CSE. However, use of the CSE Scale in digital health studies is extremely limited. As such, the main objective of the present analyses was to test whether participation in an artificial intelligence (AI)–powered digital lifestyle modification program for heart health led to improved CSE.

### Heart Health Program

The Lark Heart Health (Lark Technologies, Inc) program is a newly developed AI-powered lifestyle modification program that provides health behavior coaching to prevent and manage atherosclerotic CVD and coronary artery disease by targeting key CVD risk factors. We evaluated the acceptability and feasibility of this new program in a pilot study; a detailed study description and main outcome results for this pilot study are available elsewhere [36].

The Heart Health program targets individuals in primary prevention (ie, no history of CVD) and those in stable secondary prevention after a cardiac event. This program delivers fully remote, unlimited, real-time cardiovascular health coaching focused on digital nutrition therapy, medication adherence counseling, and personalized guidance on weight loss, physical activity, tobacco cessation, stress, and sleep. Heart Health is available to covered adult members of Lark’s health care partners via a smartphone app. Full details on the content and design of the Heart Health program are provided in previous work [36]. Full detail on Lark’s lifestyle change programs for diabetes prevention and hypertension care are provided elsewhere [37,38].

### Study Objective

Beyond the primary acceptability and feasibility aims, a secondary goal of the Heart Health pilot study was to determine whether CSE could improve early in this study. Specifically, we hypothesized that participants in the Heart Health pilot study who participated in the program for a minimum of 40 days would show improvement in CSE. This would indicate that a digital heart health program can help to improve CSE over a relatively short period, an important first step in developing scalable and accessible digital programs for improving self-efficacy among individuals at risk for CVD.

## Methods

### Study Design

This paper focuses on data from a real-world, single-arm, observational pilot study of a digital health app-based program called Lark Heart Health. The Heart Health program provides health behavior screeners and coaching to individuals at elevated risk for heart disease. The pilot study examined the feasibility of deploying screener surveys to participants of the program and the acceptability of coaching focused on building knowledge and improving self-management of atherosclerotic CVD risk. A detailed overview of study methods and primary outcomes [36] and predictors of interest in the program [39] have been reported in previously published works.

This study was 90 days in duration, during which participants received coaching on heart health and lifestyle modification. Participants could engage with the Heart Health program via (1) completing educational lessons, (2) engaging in coaching conversations with Lark’s conversational AI-powered digital coach, (3) logging meals in the app, and (4) tracking their weight using a digital smart scale that automatically synced with the app.

Additionally, study participation involved completion of baseline screener surveys and assessment of CSE at study start and again in month 2 (after 40 days in the program). We measured CSE in month 2 for several reasons. First, self-efficacy has been shown to improve after relatively short digital health interventions for lifestyle change (eg, 6 week programs) [34], particularly compared to physical health outcomes that may take months or even years to improve. Second, our study involved completing several surveys and outcome measurements that were intentionally spread out over the course of the 90 days, rather than clustering all outcome measurements at the end of this study; this approach was aimed at reducing participant burden, a crucial factor for retention in digital health programs. Third, measuring CSE early in this study enabled us to maximize the sample size of individuals actively engaging with the program who completed the survey at both time points. Between study start date and the month 2 reassessment of CSE, the Heart Health program delivered 5 weekly educational lessons focused on risk factors for heart disease, understanding cholesterol, physical activity and how it impacts blood pressure, healthy eating, and how sodium impacts blood pressure.

### Ethical Considerations

All participants provided informed consent to participate in this study through an electronic consent form. The pilot study received institutional review board approval (Advarra Pro00061694). Study personnel instituted appropriate safeguards to prevent any unauthorized use or disclosure of personal health information and implemented administrative, physical, and technical safeguards to protect the confidentiality, integrity, and availability of protected health information. Lark is compliant with HIPAA (Health Insurance Portability and Accountability Act) privacy and security rules and all applicable regulations. Lark is also SOC 2 (System and Organization Controls 2) and HITRUST (Health Information Trust Alliance) certified. As compensation for study participation, participants received a cellular smart scale and opportunities for nominal gift card incentives, as well as a Fitbit (Google LLC) upon study completion. None of these incentives were tied to completion of the CSE Scale.

### Participants and Study Flow

The research team recruited participants through a health care partner and via online recruitment through 1nHealth using marketing emails or SMS messages and printed mailers (health care partner recruitment only). Exclusion criteria for the study included: BMI <25 and ≥50; serious uncontrolled health conditions that had been active in the last 6 months; current pregnancy or plans to become pregnant within the next 6 months; recent history of a medical professional advising against participation in a healthy lifestyle program; a medical reason preventing 10 consecutive minutes of moderate physical exercise; and not having a smartphone with internet connection. As the Heart Health program focused heavily on improving health behaviors (eg, diet and exercise), the study also excluded individuals who reported regularly engaging in strenuous physical activity and individuals who did not report unhealthy dietary behaviors.

Participants enrolled in the pilot study from March 31 to September 15, 2022, using the Lark app (version 5.2.6), downloading the Heart Health app and providing a first weight delineated study enrollment. To be included in the present analyses, participants had to be enrolled until the end of this study (ie, did not withdraw from this study), complete the first CSE Scale at baseline for descriptive analyses, and complete the second CSE Scale at month 2 for the CSE improvement analyses. Completion of the CSE Scale occurred outside of the Lark app and full completion was not required for study participation.

### Data Collection and Measures

#### Participant Characteristics

We assessed basic demographic and health history characteristics at baseline using a modified version of the INTERHEART Modifiable Risk Score survey [40,41]. Items on this survey included age, sex, history of high blood pressure, tobacco use history, and typical physical activity in leisure time. Participants also provided their height and weight for calculation of BMI and the racial group they identified with.

#### CSE

Emails from the research team prompted participants to report on CSE shortly after study start (day 1‐2 of this study) and again in month 2 (after day 40 of this study) using a modified version of the CSE Scale. The CSE Scale evaluates individuals’ beliefs in their ability to manage their cardiovascular health and the challenges that come with heart disease using 13 questions in 3 dimensions [14]. The Heart Health study used 2 dimensions of the CSE Scale that are most relevant to our study sample, “control illness” and “maintain functioning*,”* resulting in a 9-item survey. We did not include the “control symptoms” dimension, as the questions are not relevant to individuals without a CVD diagnosis (ie, most individuals in the present sample) [15]. Each item is scored on a 5-point Likert scale (0=not at all confident, 1=somewhat confident, 2=moderately confident, 3=very confident, and 4=completely confident) and the total score is calculated by summing scores on each item, for a maximum score of 36. As the program did not require participants to submit the CSE Scale and permitted them to skip items, calculation of the total sum score was not possible for all study participants.

### Statistical Analysis

We conducted all statistical analyses with RStudio (version 2022.07.0; R Foundation). Continuous variables were normally distributed. The first set of analyses examined relationships between baseline participant characteristics and baseline CSE Scale score. Specifically, we tested the relationship of baseline CSE with demographic characteristics (age, sex, and race), health status and health history characteristics (high blood pressure, cardiac event, or CVD diagnosis—secondary prevention, and type II diabetes), and health behaviors (tobacco use and typical physical activity in leisure time). We used bivariate regressions for continuous variables (age and BMI), independent samples *t* tests for categorical variables with 2 levels (sex and health history variables), and 1-way ANOVAs for categorical variables with 3 levels (race and health behaviors). Baseline CSE analyses included participants who completed the baseline CSE Scale (n=341).

To address the primary study objective, the second set of analyses tested whether there was a statistically significant change in CSE from baseline to month 2, using paired *t* tests (*P*<.05). We first tested whether there was a significant change in the CSE sum score and then whether there was improvement in each of the 9 individual items on the CSE. These analyses included all participants who completed both the baseline CSE and month 2 CSE scales (n=273).

## Results

**Figure 1.**
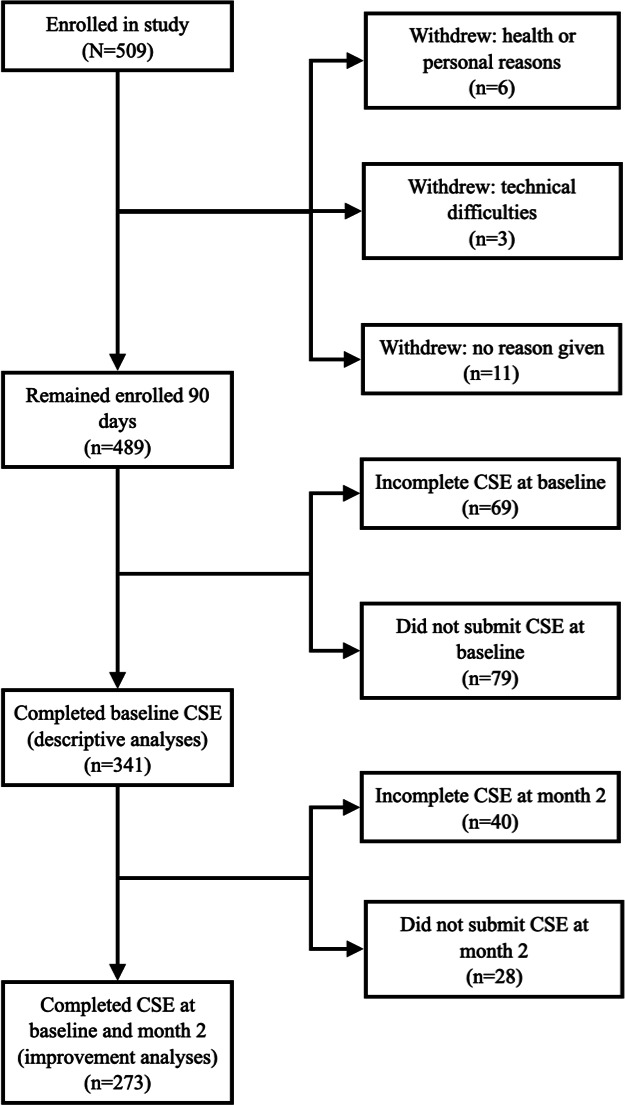
Flowchart of participants showing enrollment and inclusion in the analytic sample. CSE: cardiac self-efficacy.

Figure 1 shows the flow of participants through this study.

### Descriptive Statistics

We assessed descriptive statistics for baseline characteristics of participants who completed the baseline CSE Scale (n=341) and participants who completed both the baseline and month 2 CSE (n=273); both samples are shown in Table 1, as we conducted baseline descriptive analyses on the larger sample and improvement analyses on the smaller subsample (Table 1). As shown by the table, the 2 sets of participants were very similar with respect to key characteristics. Across both sets participants were, on average, aged 60 years (range*=*41‐76 years) and classified as obese class I (BMI range=25‐49). Just over 60% (n=207) of the participants were female, had a history of high blood pressure, and were never smokers. Approximately three-quarters of each sample reported typically being either mainly sedentary or engaging in low-effort mild exercise in their leisure time.

**Table 1.** Baseline characteristics of participants.

		Baseline CSE^a^ complete (n=341)	Baseline and month 2 CSE complete (n=273)
Age (years), mean (SD)		60 (10.1)	59.3 (10.1)
Sex (female), n (%)		207 (60.7)	167 (61.2)
Race, n (%)			
	Black	26 (7.6)	22 (8.1)
	White	241 (70.7)	192 (70.3)
	Other	70 (20.5)	57 (20.9)
Recruitment source (health partner), n (%)		207 (60.7)	154 (56.4)
Baseline BMI, mean (SD)		32.8 (5.8)	32.9 (5.9)
High blood pressure history, n (%)		198 (58.1)	162 (59.3)
History of CVD^b^ diagnosis or event, n (%)		41 (12.0)	34 (12.5)
Type II diabetes history, n (%)		52 (15.2)	37 (13.6)
Tobacco use status, n (%)			
	Never	203 (59.5)	171 (62.6)
	Former	110 (32.3)	82 (30.0)
	Current	27 (7.9)	19 (6.9)
Physical activity in leisure time, n (%)			
	Mainly sedentary	121 (35.5)	94 (34.4)
	Mild exercise, low effort	145 (42.5)	116 (42.5)
	Moderate exercise	72 (21.1)	61 (22.3)
Baseline CSE score, mean (SD)		25.1 (7.3)	25.1 (7.3)

a CSE: cardiac self-efficacy.

b CVD: cardiovascular disease.

### Relationships Between Baseline CSE Scores and Participant Characteristics

Next, we examined relationships between baseline participant characteristics and baseline CSE Scale scores for all participants who completed the baseline CSE Scale. Of the demographic characteristics, age was significantly associated with CSE, such that CSE increased with age (n=341, β=.12*,* SE=0.04, *P*=.03). There was no statistically significant difference in baseline CSE by sex (*t*_338_=0.19, *P*=.43) or racial group (*F*_340_=2.72, *P*=.07).

Among the health history characteristics, participants with a history of type II diabetes tended to have higher baseline CSE (mean 27.48, SD 6.47, n=52) compared to participants without diabetes history (mean 24.71, SD 7.42, n=289; *t*_77_=2.78, *P*=.003). There was no difference in baseline CSE between participants with a history of high blood pressure and those without (*t*_322_=0.91, *P*=.36) or participants in primary prevention versus secondary prevention (*t*_50_=0.57, *P*=.57). There was also a significant association between BMI and CSE, with lower BMI individuals reporting higher CSE (n=341, β=−.11, SE=0.07, *P*=.047).

For the health behavior variables, there was no difference in CSE based on tobacco use status (*F*_339_=1.61, *P*=.20) or typical physical activity in leisure time (*F*_337_=1.51, *P*=.22)

### Change in CSE From Baseline to Month 2

In testing for changes in the CSE from baseline to month 2, we found a significant increase in the overall sum score on the CSE Scale from baseline to month 2 (Table 2). There were also significant increases in each individual item on the CSE (all *P*<.001). The individual CSE items with the greatest increases were “confidence in knowing when to call or visit the doctor,” “confidence in knowing how much physical activity is good for you,” and “confidence that you can get regular aerobic exercise.*”*

**Table 2.** Changes in CSE^a^ score from baseline to month 2 (n=273)^b^. The baseline CSE survey was surfaced to participants on day 2, and the month 2 CSE survey was deployed on day 40.

		Baseline, mean (SD)	Month 2, mean (SD)	Baseline to month 2, raw change	Baseline to month 2, change in %	*t* test (*df*)	*P* value
How confident are you that you know…							
	When to call or visit your doctor about your heart disease?	2.7 (1.1)	3.1 (0.9)	+0.5	+17	7.9 (272)	<.001
	How to make your doctor understand your concerns about your heart?	2.8 (1.1)	3.1 (0.9)	+0.3	+10.7	5.1 (272)	<.001
	How to take your cardiac medications?	3.1 (1.1)	3.4 (0.9)	+0.4	+11.4	5.7 (272)	<.001
	How much physical activity is good for you?	2.7 (1.1)	3.2 (0.8)	+0.5	+16.3	7.7 (272)	<.001
How confident are you that you can...							
	Maintain your social activities?	2.8 (1)	3.2 (0.8)	+0.4	+13	6.4 (272)	<.001
	Maintain your usual activities at home with your family?	3 (1)	3.3 (0.8)	+0.2	+7.7	4.2 (272)	<.001
	Maintain your usual activities at work?	2.9 (1.1)	3.2 (0.9)	+0.3	+10.7	4.8 (272)	<.001
	Maintain your sexual relationship with your spouse?	2.6 (1.3)	2.9 (1.1)	+0.3	+12.3	4.3 (272)	<.001
	Get regular aerobic exercise?	2.5 (1.2)	2.9 (1.1)	+0.5	+19	6.4 (272)	<.001
Overall sum score on the CSE		25.07 (7.3)	28.3 (6.3)	+3.2	+12.9	9.2 (272)	<.001

a CSE: cardiac self-efficacy.

b n=273 participants submitted the cardiac self-efficacy with all items completed.

## Discussion

### Summary and Interpretation of Results

These analyses examined improvement in CSE as a secondary goal in a digital lifestyle modification program for heart health focused on AI-supported health coaching, educational lessons, behavioral tracking, and weight management. We found that the overall CSE Scale score improved significantly from baseline to month 2 of the program. Moreover, mean scores on each individual item of the CSE increased significantly over this short period, with the largest increases in patients’ confidence in knowing when to contact their doctor about their heart health (+17%) and items related to confidence in knowing how much physical activity to do (+16.3%) and getting regular exercise (+19%). The results of these secondary analyses of the Heart Health program pilot study support the assertion that digital health programs can play an important role in increasing self-efficacy to better manage their health over a relatively short period.

This study is the first digital health study we are aware of to show improvements in CSE scores through participation in a digital lifestyle modification program for heart health. There is evidence that in-person lifestyle change programs for CVD can improve CSE [25,26], and literature showing that digital interventions can increase general self-efficacy [34,35]. These results are consistent with results from 2 prior studies with cardiac patients showing that in-person intervention programs for CVD management can improve CSE scores; however, these in-person programs involved intensive, human-led interventions that required significant staffing and economic resources [25,26].

One recent study using a 60-day text message intervention implemented in patients recently discharged after acute coronary syndrome had no significant effect on the CSE [42]. The researchers implemented an automated 1-way text message campaign through which patients received messages about self-management, healthy living, and follow-up care. There are many key differences in the study design by Ross et al [42] compared to our study, but it is possible that we found improvements in CSE because the Heart Health program involves 2-way interaction with AI-supported coaching and active behavior tracking, rather than a 1-way messaging system.

It is also notable that 2 of the CSE items with the biggest improvement from baseline to month 2 were centered on confidence related to physical activity: “confidence in knowing how much physical activity is good for you” (+16%), and “confidence that you can get regular aerobic exercise*”* (+19%). Education on physical activity is a key component of the Heart Health program, with an educational lesson on Physical Activity for Low Blood Pressure delivered during the third weekly lesson in the program. The Heart Health digital coach also provides ongoing interactive coaching on physical activity, encouraging participants to log their physical activity in the app. As such, the present results suggest that the education and coaching in the first 2 months of the Heart Health program support improvements in confidence related to physical activity. Similarly, prior work has shown that digital health interventions focused on physical activity can improve exercise-related self-efficacy over 24 weeks [43]. Future research in this area could examine whether improvements in CSE Scale score during the first 2 months of the program predict longitudinal increases in physical activity.

Beyond the primary results of these analyses, we also found several relationships between baseline CSE score and participant characteristics. Specifically, we found that older age, lower BMI, and having a history of type II diabetes were all associated with having higher CSE scores at baseline. Importantly, a significant body of literature has shown that higher self-efficacy among older persons is associated with a range of positive health benefits, including increased self-care, better health behaviors, increased energy, and decreased pain and discomfort [44,45]. This is particularly relevant to our study, as participants in this sample tended to be older adults, with a mean age of 60 years and up to 76 years. Notably, the broader literature indicates that self-efficacy and closely related constructs, such as self-esteem, tend to increase with age due to increasing knowledge and maturity, greater control over life circumstances, higher likelihood of occupying positions of power and status, and improved coping resources and processes [46]. Indeed, a large meta-analysis of 164,868 participants showed that self-esteem tends to increase steadily with age, reaching peak levels between ages 60‐70 before declining slightly after age 70 and steeply after age 90 [47].

Our finding that lower BMI was associated with high CSE scores aligns with prior work showing that cardiac patients with lower BMI also tended to have higher CSE [48] and with population-based research showing lower health-related self-efficacy among individuals with higher BMI [49]. This relationship may be due to individuals with higher BMI having a history of difficulty managing their weight and lower self-esteem [50], factors that are closely related to self-efficacy.

The link between the history of type II diabetes and baseline CSE is also consistent with prior literature showing that patients with a history of chronic disease diagnosis tend to have higher CSE [48,51]. This relationship is likely due to these individuals already having some experience with managing a health condition and potentially receiving prior education on managing cardiometabolic conditions. However, it is surprising that a history of high blood pressure and history of cardiac events or CVD diagnoses were not associated with differences in baseline CSE in this study’s sample.

### Limitations and Future Directions

There are several limitations to our results. First, data for these secondary analyses came from an observational pilot study focused on the acceptability and feasibility of a new digital heart health program. As such, these results should be considered preliminary, rather than conclusive. We were also unable to compare these results to a control group, and causal conclusions are limited. Additionally, participants could opt out of any survey, including the CSE survey, if they did not wish to complete it. As a result, we could only include individuals in these analyses if they submitted a complete CSE Scale. Completion of the CSE Scale also occurred outside of the Lark app to be used for pilot study purposes only. Thus, the sample may be biased toward participants who were more engaged in the program or more likely to check their emails. The CSE Scale is also limited as it includes questions about confidence in work settings and in marital life, which may not apply to all participants. Finally, this digital intervention was limited to those with a smartphone with internet access who had sufficient digital literacy to use the Lark app. As a result, this may exclude individuals of lower socioeconomic status. For instance, the 2024 Pew Research Center data showed that, although 90% of US adults have a smartphone, individuals in the lowest income bracket (US <$30,000/year) have lower rates of smartphone ownership (79%) compared to those in higher income brackets (US >$100,000/year; 98%) [52].

The pilot study results presented here provide a foundation for several lines of future work. For instance, future work including a control group can provide causal evidence for the impact of the Heart Health program on CSE. Additionally, a key future direction for this study will be assessing whether increased CSE predicts longitudinal clinical outcomes. Given that the duration of the pilot study was only a short period of 90 days, we did not expect to see a meaningful relationship between improved CSE Scale scores and clinical outcomes. This is particularly true given that we measured CSE improvements nearly halfway through the program. Future research could examine longitudinal changes in CVD risk factors, such as blood pressure or cholesterol levels. Based on the literature, we would expect that improvements in CSE would predict improved cardiovascular outcomes.

### Conclusions

In summary, the present analyses indicate that participants in a digital lifestyle modification program for heart health showed significant improvements in CSE within 2 months. This work adds to the growing literature examining ways to improve health-related self-efficacy for the prevention and management of chronic disease and long-term health benefits.

## Abbreviations

AI: artificial intelligence
CSE: cardiac self-efficacy
CVD: cardiovascular disease
HIPAA: Health Insurance Portability and Accountability Act
HITRUST: Health Information Trust Alliance
SOC 2: System and Organization Controls 2
